# The protective effect of licofelone on experimental osteoarthritis is correlated with the downregulation of gene expression and protein synthesis of several major cartilage catabolic factors: MMP-13, cathepsin K and aggrecanases

**DOI:** 10.1186/ar1788

**Published:** 2005-07-19

**Authors:** Jean-Pierre Pelletier, Christelle Boileau, Martin Boily, Julie Brunet, François Mineau, Changshen Geng, Pascal Reboul, Stefan Laufer, Daniel Lajeunesse, Johanne Martel-Pelletier

**Affiliations:** 1 Osteoarthritis Research Unit, University of Montreal Hospital Centre, Notre-Dame Hospital, Montreal, Quebec, Canada; 2 Department of Pharmaceutical Chemistry/Medicinal Chemistry, Eberhard-Karls-University Tübingen, Institute of Pharmacy, Tübingen, Germany

## Abstract

This study sought to evaluate the levels of mRNA expression and protein synthesis of MMP-13, cathepsin K, aggrecanase-1 (ADAMTS-4), aggrecanase-2 (ADAMTS-5) and 5-lipoxygenase (5-LOX) in cartilage in the experimental anterior cruciate ligament (ACL) dog model of osteoarthritis (OA), and to examine the effects of treatment with licofelone, a 5-lipoxygenase (LOX)/cyclooxygenase (COX) inhibitor, on the levels of these catabolic factors. Sectioning of the ACL of the right knee was performed in three experimental groups: group 1 received no active treatment (placebo group); and groups 2 and 3 received therapeutic concentrations of licofelone (2.5 or 5.0 mg/kg/day orally, respectively) for 8 weeks, beginning the day following surgery. A fourth group consisted of untreated dogs that were used as normal controls. Specimens of cartilage were selected from lesional areas of OA femoral condyles and tibial plateaus, and were processed for real-time quantitative PCR and immunohistochemical analyses. The levels of MMP-13, cathepsin K, ADAMTS-4, ADAMTS-5 and 5-LOX were found to be significantly increased in OA cartilage. Licofelone treatment decreased the levels of both mRNA expression and protein synthesis of the factors studied. Of note was the marked reduction in the level of 5-LOX gene expression. The effects of the drug were about the same at both tested dosages. *In vivo *treatment with therapeutic dosages of licofelone has been found to reduce the degradation of OA cartilage in experimental OA. This, coupled with the results of the present study, indicates that the effects of licofelone are mediated by the inhibition of the major cartilage catabolic pathways involved in the destruction of cartilage matrix macromolecules. Moreover, our findings also indicate the possible auto-regulation of 5-LOX gene expression by licofelone in OA cartilage.

## Introduction

Along with the graying of the world's population, osteoarthritis (OA), the most common form of arthritis, is becoming an increasingly significant medical and financial burden. In this context, the clear need for a better understanding of the disease process has rendered undeniable the importance of finding drugs that can reduce or stop its progression.

Recent studies have revealed new and interesting information regarding the role played by eicosanoids in the pathophysiology of arthritic diseases, including OA [1-6]. For instance, leukotriene-B_4 _(LTB_4_) has proven to be an important regulating factor in the synthesis of IL-1β by OA synovium [6-8]. Both *in vitro *and *in vivo *studies have demonstrated that the excess production of IL-1β in OA tissue is a key factor in its destruction and in the progression of the disease itself [1,9]. The endogenous production of LTB_4 _in OA synovium is a crucial element in the upregulation of IL-1β synthesis in this tissue [8]. The synthesis of LTB_4_, and subsequently of IL-1β, can be significantly increased by non-steroidal anti-inflammatory drugs (NSAIDs) [10,11]. It has been hypothesized that this could be related to a 'shunt' of the arachidonic acid cascade from the cyclooxygenase (COX) to the lipoxygenase (LOX) pathway [2]. These findings could help explain how some NSAIDs accelerate the progression of clinical OA [12]. A recent study from our laboratory has demonstrated that, in *in vivo *experimental OA, licofelone, a drug that can inhibit both the COX and 5-LOX pathways, was capable of reducing the development of OA structural changes while simultaneously reducing the synthesis of LTB_4 _and IL-1β by the OA synovium [6]. These findings are in strong support of the *in situ *role played by LTB_4 _in the structural changes that occur in OA.

The progression of the structural changes that occur during the course of the disease is related to a number of complex pathways and mechanisms, among which the excess production of proteolytic enzymes that can degrade the cartilage matrix and soft tissues surrounding the joint is believed to be of particular importance [1]. The degradation of the OA cartilage matrix has been shown to be related to the excess synthesis of a large number of proteases and, more particularly, to that of the matrix metalloproteinases (MMPs) and thiol-dependent families. Among the MMPs, two collagenases, MMP-1 and MMP-13, have been the subject of extensive investigation and were found likely to be the primary enzymes involved in the breakdown of type II collagen in OA cartilage [13]. Cathepsin K, a thiol-dependent enzyme that works preferentially under acidic pH conditions, has also been demonstrated to be synthesized by OA chondrocytes and is likewise believed to play an important role in the breakdown of the OA cartilage collagen network [14] as well as the aggrecans, and thus likely involved in degrading the cartilage extracellular matrix. The mechanisms involved in the degradation of the aggrecans in OA cartilage have also been extensively explored and studied, which has led to the identification of a number of proteolytic enzymes that can specifically degrade aggrecans [15]. Comprehensive investigation has indicated that the MMPs, including MMP-13, aggrecanase-1 (a disintegrin and metalloproteinase with thrombospondin motifs (ADAMTS)-4) and aggrecanase-2 (ADAMTS-5), are the proteolytic enzymes that seem the most likely to be involved in the degradation of aggrecans in OA cartilage [16,17].

The present study is an extension of previous ones that investigated the mechanisms by which licofelone, a dual inhibitor of 5-LOX and COXs, can reduce the development of experimental OA. This study focuses on the *in situ *effect of licofelone on the gene expression and protein synthesis of the major collagenolytic enzymes (MMP-13 and cathepsin K) and aggrecan-degrading proteases (ADAMTS-4 and ADAMTS-5) in OA cartilage using the experimental anterior cruciate ligament (ACL) model in dogs. The level of 5-LOX in OA cartilage as well as the drug treatment effects were also explored.

## Materials and methods

### Experimental groups

Specimens were obtained from different experimental groups, including some that had been included in previous studies [6,18]. Adult crossbred dogs of 2 to 3 years of age, weighing 20 to 25 kg each, were used in the study. The surgical sectioning of the ACL of the right knee was performed through a stab wound, as previously described [6]. Prior to surgery, the animals were intravenously anesthetized with pentobarbital sodium (25 mg/kg) and intubated. After surgery, the dogs were kept in animal care facilities for one week, and were then sent to a housing farm. Dogs were housed in a large pen in which they could exercise *ad libitum *under supervision to ensure that they were bearing weight on the operated knee. The University of Montreal Hospital Centre Research Ethics Committee at the Notre-Dame Hospital approved the protocol.

The dogs were separated into four experimental groups: group 1 (n = 7) consisted of OA operated dogs that received the placebo (encapsulated methylcellulose); group 2 (n = 7) of OA operated dogs that received encapsulated licofelone (2.5 mg/kg/day orally) (Merckle GmbH, Ulm, Germany); group 3 (n = 7) of OA operated dogs that received encapsulated licofelone (5.0 mg/kg/day orally); and group 4 (n = 6) of normal unoperated dogs (n = 6) that received no treatment. All treatments began the day after surgery. The dosages were selected on the basis of those given to patients for the treatment of symptomatic OA [6]. Licofelone was administered twice daily (at 8 a.m. and 4 p.m.) with food to a total dosage of 2.5 or 5.0 mg/kg. All dogs were sacrificed 8 weeks after surgery, including group 4, which was used as a control group. Morphologic changes in OA dogs have already been reported [6].

### Specimen selection and preparation

As previously described [6,19], a full-thickness section of articular cartilage was removed from the lesional areas of the femoral condyles and tibial plateaus of the placebo-treated OA dogs, and from the OA dogs treated with 2.5 mg/kg/day or 5.0 mg/kg/day of licofelone. Specimens were also obtained from equivalent anatomical sites in the normal dogs. The specimens were embedded in paraffin and processed for immunohistological studies.

### Histologic grading

Histologic evaluation was performed on sagittal sections of cartilage from the lesional areas of femoral condyles and tibial plateaus as described [6]. Specimens were fixed in TissuFix #2 (Chaptec Inc., Montreal, QC, Canada) for 24 h, then embedded in paraffin. Serial sections (5 μm) of paraffin-embedded specimens were stained with safranin-O. The severity of the OA lesions was graded on a scale of 0–14 by two independent observers using the histologic/histochemical scale of Mankin *et al*. [20]. The scale evaluates the loss of safranin-O staining (scale 0–4), cellular changes (scale 0–3), invasion of the tide mark by blood vessels (scale 0–1) and structural changes (scale 0–6, where 0 = normal cartilage structure and 6 = erosion of the cartilage down to the subchondral bone). Scoring was based on the most severe histologic changes within each cartilage section.

### Immunohistochemistry

Cartilage specimens from femoral condyles and tibial plateaus (n = 5 per group) were processed for immunohistochemical analysis, as previously described [6,18,19]. Specimens were fixed in TissuFix #2 (Chaptec Inc.) for 24 h, then embedded in paraffin. Sections (5 μm) of paraffin-embedded specimens were placed on Superfrost Plus slides (Fisher Scientific, Nepean, ON, Canada), deparaffinized in xylene, rehydrated in a reverse-graded series of ethanol, and preincubated with chondroitinase ABC 0.25 units/ml (Sigma-Aldrich Canada, Oakville, ON, Canada) in PBS pH 8.0 for 60 minutes at 37°C. The specimens were subsequently washed in PBS, incubated in 0.3% Triton X-100/PBS for 30 minutes, and then placed in 3% hydrogen peroxide/PBS for 15 minutes. Slides were further incubated with a blocking serum (Vectastain ABC kit; Vector Laboratories Inc., Burlingame, CA, USA) for 60 minutes, after which they were blotted and then overlaid with the primary polyclonal goat antibody against collagenase-3 (MMP-13) (15 μg/ml; R&D Systems, Minneapolis, MN, USA); polyclonal goat antibody against cathepsin K (1 μg/ml; Santa Cruz, Santa Cruz, CA, USA); polyclonal rabbit antibody against ADAMTS-4 (RP1ADAMTS-4) or ADAMTS-5 (RP1ADAMTS-5) (10 μg/ml; Triple Point Biologics Inc., Forest Grove, OR, USA); or rabbit antiserum against 5-LOX (dilution 1:50; Cayman Chemical, Ann Arbor, MI, USA) for 18 h at 4°C in a humidified chamber. The antibodies against MMP-13, ADAMTS-4 and ADAMTS-5 recognized both the pro- and active forms of the enzyme. Each slide was washed three times in PBS (pH 7.4) and stained using the avidin-biotin complex method (Vectastain ABC kit), which entails incubation in the presence of the biotin-conjugated secondary antibody for 45 minutes at room temperature, followed by the addition of the avidin-biotin-peroxidase complex for 45 minutes. All incubations were carried out in a humidified chamber at room temperature and the colour was developed with 3,3'-diaminobenzidine (Vector Laboratories, Inc.) containing hydrogen peroxide. Slides were counterstained with eosin.

To determine the specificity of staining, different control procedures were employed according to the same experimental protocol: first, the use of adsorbed immune serum (1 h, 37°C) with a 20-fold excess of human recombinant for MMP-13 protein (R&D Systems) and for 5-LOX protein (Cayman Chemical), or human blocking peptide for cathepsin K (Santa Cruz) and ADAMTS-4 (Triple Point Biologics Inc.) (the peptide for ADAMTS-5 was not commercially available); second, omission of the primary antibody; and third, substitution of the primary antibody with an autologous pre-immune serum. The results of control experiments for MMP-13 and cathepsin K have already been published [18] and showed only background staining.

### Immunohistomorphometric analysis

Several sections were made from each block of cartilage, and three non-consecutive representative sections from each specimen were processed for immunohistochemical analysis. Each section was examined under a light microscope (Leitz Orthoplan; Wild Leitz, St. Laurent, QC, Canada) and photographed with a CoolSNAP cf Photometrics camera (Roper Scientific, Rochester, NY, USA). The different antigen levels were quantified using a method modified from our previously published studies [6,21]. by determining the number (percentage) of chondrocytes that stained positive. Each section was divided into six macroscopic fields (three in superficial and three in the deep zones of cartilage) (×40; Leitz Diaplan). The superficial zone of cartilage corresponds to the superficial and to the upper intermediate layers. The deep zone of cartilage corresponds to the lower intermediate and the deep layers. The results from the six fields were averaged for each section. The total number of cells and the number of cells that stained positive for the specific antigen were determined. The results were expressed as the percentage of cells that stained positive for the antigen (cell score), with the maximum score being 100%. Each slide was subjected to a double-blind evaluation, which resulted in a variation of less than 5%. For the purposes of statistical analysis, the data obtained for each specimen (mean score of three sections) were considered independent.

### Real-time quantitative PCR analysis

#### Extraction of total RNA from cartilage

Total RNA was extracted directly from the cartilage. The cartilage from the condyles and the plateaus (0.5–1.0 g) was pooled to allow for the processing of a sufficient amount of tissue for RNA extraction. Cartilage was suspended in a TRIzol buffer (Invitrogen; Life Technologies, Burlington, ON, Canada) and processed as previously described [22]. The purified RNA was quantified by spectrophotometry.

#### PCR analysis

The quantification of gene expression for MMP-13, cathepsin K, 5-LOX, ADAMTS-4, and ADAMTS-5 was determined by real-time quantitative PCR with the GeneAmp^® ^5700 Sequence Detection System (Applied Biosystems, Foster City, CA, USA) using the Quantitect Sybr Green PCR kit (Qiagen Inc., Mississauga, ON, Canada), as previously described [23].

The oligonucleotides used for PCR studies are described in Table 1. The data were collected and processed with GeneAmp^® ^5700 SDS software and given as a threshold cycle (C_t_). Plasmid DNA containing the target gene sequences was used to generate standard curves. A DNA standard curve for each gene was prepared and used in quantitative PCR reactions. The C_t _was then converted to a number of molecules, and the value for each sample was calculated as the ratio of the number of molecules of the target gene to the number of molecules of glyceraldehyde-3-phosphate dehydrogenase (GAPDH) gene. The primer efficiencies for the test genes were the same as those for the GAPDH gene.

### Statistical analysis

Unless otherwise specified, values are expressed as the median with the range in parentheses. Statistical analysis was performed using the Mann-Whitney U test. Correlations between the histologic grade and the cell score were analyzed using a linear regression test. Statistical analysis was performed using the parametric (Pearson) linear correlation test. P-values ≤ 0.05 were considered significant.

## Results

### Histologic analysis

Cartilage from normal controls had normal microscopic appearance. Specimens from the OA group presented typical OA changes with a Mankin score of 5.1 (3–11) and a safranin-O score of 1 (0–4). Specimens from licofelone-treated groups had a Mankin score of 3.5 (0–10) and a safranin-O score of 0.4 (0–3) with the 2.5 mg dosage and a Mankin score of 4.2 (1.5–6.5) and a safranin-O score of 0.3 (0–1.5) with the 5.0 mg dosage.

### MMP-13 gene expression and protein synthesis

**Figure 1:**
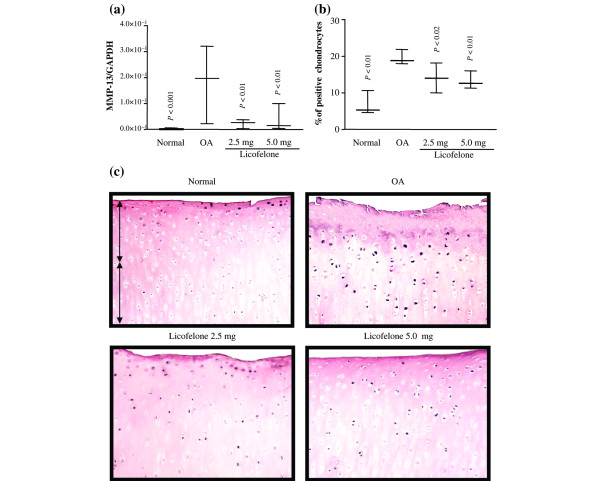
MMP-13 gene expression and protein synthesis. **(a) **mRNA levels, as determined by real-time quantitative PCR analysis as described in Materials and methods. **(b) **Morphometric analysis of MMP-13 immunostaining. **(a, b) **Data are expressed as median and range and are presented as box plots, where the boxes represent the 1^st ^and 3^rd ^quartiles, the line within the box represents the median, and the lines outside the box represent the spread of values. P-values were compared to the placebo group (OA) using the Mann-Whitney U test. **(c) **Representative MMP-13 immunohistochemical sections of tibial plateaus. Superficial (superfical and upper intermediate layers) and deep (lower intermediate and deep layers) zones of cartilage are indicated on the picture with arrows. No specific staining was detected in the OA cartilage with immunoabsorbed serum (data not shown) (original magnification × 250). GAPDH, glyceraldehyde-3-phosphate dehydrogenase; MMP, matrix metalloproteinase.

PCR analysis found a marked and significant increase in the expression of mRNA for MMP-13 in OA cartilage compared to normal (Fig. 1). Immunohistochemical analysis revealed that the increased synthesis of MMP-13 was mainly found throughout the tissue, as previously reported [18]; the controls were negative (data not shown). A good correlation exists between the mRNA and protein levels. At the two dosages tested, the licofelone treatment significantly reduced the levels of both MMP-13 mRNA expression and the protein to an approximately similar extent.

### Cathepsin K gene expression and protein synthesis

**Figure 2:**
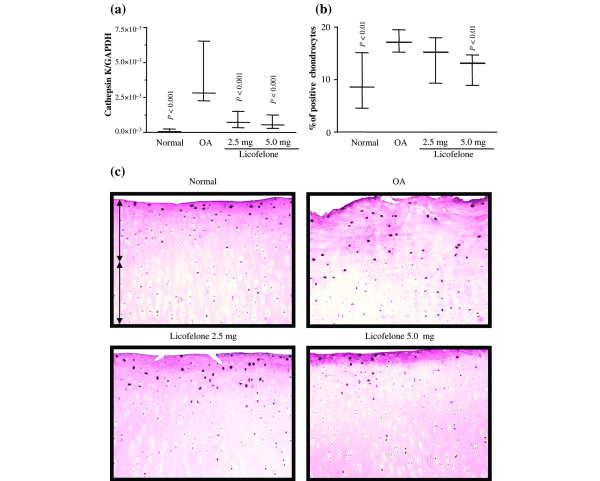
Cathepsin K gene expression and protein synthesis. **(a) **mRNA levels, as determined by real-time quantitative PCR analysis as described in Materials and methods. **(b) **Morphometric analysis of cathepsin K immunostaining. **(a, b) **Data are expressed as median and range and are presented as box plots, where the boxes represent the 1^st ^and 3^rd ^quartiles, the line within the box represents the median, and the lines outside the box represent the spread of values. P-values were compared to the placebo group (OA) using the Mann-Whitney U test. **(c) **Representative cathepsin K immunohistochemical sections of tibial plateaus. Superficial (superfical and upper intermediate layers) and deep (lower intermediate and deep layers) zones of cartilage are indicated on the picture with arrows. No specific staining was detected in the OA cartilage with immunoabsorbed serum (data not shown) (original magnification × 250). GAPDH, glyceraldehyde-3-phosphate dehydrogenase.

The levels of both the gene expression and the protein of cathepsin K were significantly increased in OA cartilage, compared to normal cartilage (Fig. 2). These two levels were also well correlated. Immunohistochemical staining showed that the enzyme was found to be preferentially located in the superficial zone of the OA cartilage, as previously reported [18]. The controls were found to be negative (data not shown). Treatment with licofelone at both concentrations reduced the levels of mRNA expression and protein synthesis of cathepsin K. The effect was similar at both of the tested dosages for gene expression and more pronounced at the highest dosage tested for the level of the enzyme *per se*.

### ADAMTS-4 and ADAMTS-5 gene expression and protein synthesis

**Figure 3:**
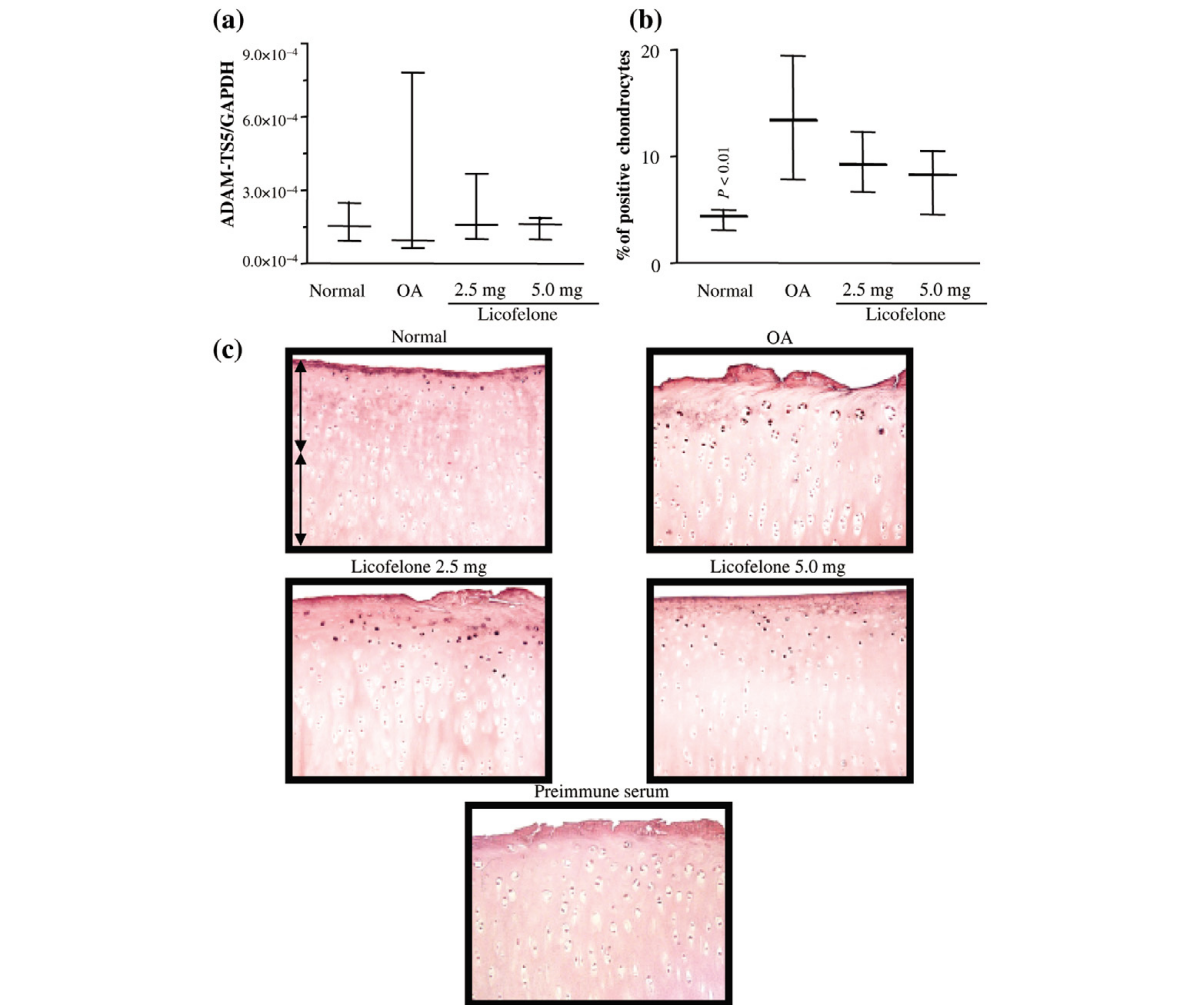
ADAMTS-5 gene expression and protein synthesis. **(a) **mRNA levels, as determined by real-time quantitative PCR analysis as described in Materials and methods. **(b) **Morphometric analysis of ADAMTS-5 immunostaining. **(a, b) **Data are expressed as median and range and are presented as box plots, where the boxes represent the 1^st ^and 3^rd ^quartiles, the line within the box represents the median, and the lines outside the box represent the spread of values. P-values were compared to the placebo group (OA) using the Mann-Whitney U test. **(c) **Representative ADAMTS-5 immunohistochemical sections of tibial plateaus. Superficial (superfical and upper intermediate layers) and deep (lower intermediate and deep layers) zones of cartilage are indicated on the picture with arrows. No specific staining was detected in the OA cartilage with immunoabsorbed serum (data not shown) (original magnification × 250). GAPDH, glyceraldehyde-3-phosphate dehydrogenase.

**Figure 4:**
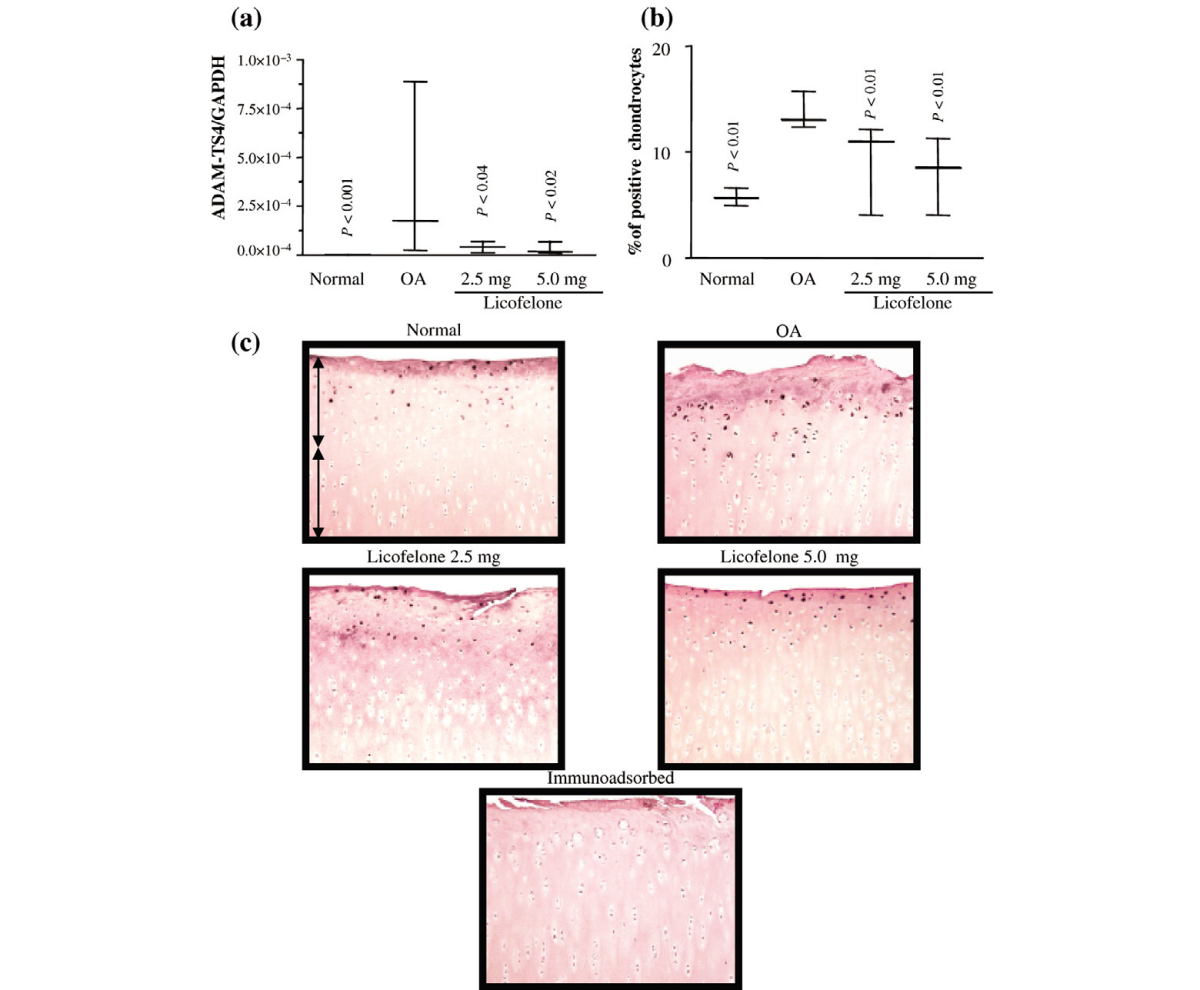
ADAMTS-4 gene expression and protein synthesis. **(a) **mRNA levels, as determined by real-time quantitative PCR analysis as described in Materials and methods. **(b) **Morphometric analysis of ADAMTS-4 immunostaining. **(a, b) **Data are expressed as median and range and are presented as box plots, where the boxes represent the 1^st ^and 3^rd ^quartiles, the line within the box represents the median, and the lines outside the box represent the spread of values. P-values were compared to the placebo group (OA) using the Mann-Whitney U test. **(c) **Representative ADAMTS-4 immunohistochemical sections of tibial plateaus. Superficial (superfical and upper intermediate layers) and deep (lower intermediate and deep layers) zones of cartilage are indicated on the picture with arrows. No specific staining was detected in the OA cartilage with immunoabsorbed serum (data not shown) (original magnification × 250). GAPDH, glyceraldehyde-3-phosphate dehydrogenase.

The level of gene expression of ADAMTS-5 in OA cartilage determined by PCR analysis was highly variable and, although sometimes higher than that in normal cartilage, the differences did not reach statistical significance (Fig. 3). The results were somewhat similar with regards to the immunohistochemical analysis. The staining showed that the enzyme was in the chondrocytes mainly located in the superficial zone; some matrix staining was also observed. The protein level of ADAMTS-5 in OA cartilage was found to be significantly higher than normal; the controls were found to be negative and showed only background staining. Treatment with licofelone had little effect on the level of its gene expression or on the level of protein. In contrast, the level of expression of mRNA for ADAMTS-4 was found to be significantly increased in OA cartilage compared to normal (Fig. 4). This was also reflected in the immunohistochemical analysis, in which an increased level of the enzyme was found more particularly in the superficial layers. The level of the enzyme was found to be significantly decreased by licofelone treatment at both the tested dosages.

### 5-LOX gene expression and protein synthesis

**Figure 5:**
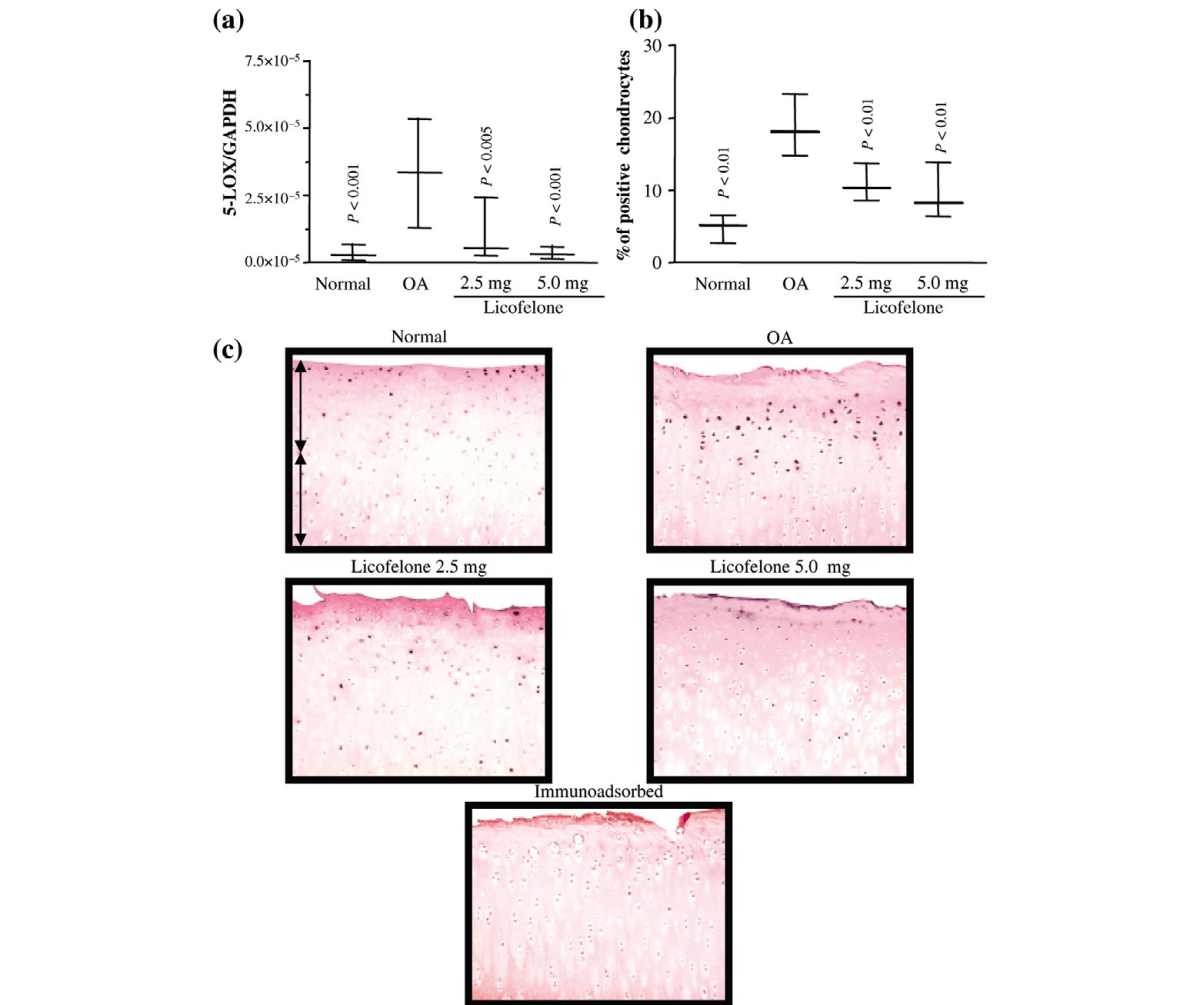
5-LOX gene expression and protein synthesis. **(a) **mRNA levels, as determined by real-time quantitative PCR analysis as described in Materials and methods. **(b) **Morphometric analysis of 5-LOX immunostaining. **(a, b) **Data are expressed as median and range and are presented as box plots, where the boxes represent the 1^st ^and 3^rd ^quartiles, the line within the box represents the median, and the lines outside the box represent the spread of values. P-values were compared to the placebo group (OA) using the Mann-Whitney U test. **(c) **Representative 5-LOX immunohistochemical sections of tibial plateaus. Superficial (superfical and upper intermediate layers) and deep (lower intermediate and deep layers) zones of cartilage are indicated on the picture with arrows. No specific staining was detected in the OA cartilage with immunoabsorbed serum (data not shown) (original magnification × 250). GAPDH, glyceraldehyde-3-phosphate dehydrogenase.

Although the level of gene expression of 5-LOX in normal cartilage was very low, as demonstrated by quantitative PCR analysis (Fig. 5), it showed a marked and significant increase in OA cartilage. There was a good correlation between these results and those from immunohistochemistry, which also showed a marked and significant increase in the level of the enzyme that was mainly located in the superficial zone of OA cartilage. The controls were negative. At both of the tested dosages, licofelone treatment significantly reduced the level of gene expression and protein synthesis of the enzyme to a similar extent. There was also a correlation between the reduction in the mRNA and protein levels.

### Correlation analysis: Mankin score, safranin-O and cell score

In specimens from OA dogs, a positive and significant correlation was found between the Mankin score or the safranin-O staining score and the chondrocyte cell score for ADAMTS-4 (r = 0.50, p = 0.005 for the Mankin score, and r = 0.59, p = 0.001 with safranin-O) and ADAMTS-5 (r = 0.52, p = 0.005 for the Mankin score, and r = 0.47, p = 0.019 with safranin-O) staining. A positive and significant correlation was also found between the Mankin score or the safranin-O staining score and the chondrocyte cell score for 5-LOX (r = 0.45, p = 0.01 for the Mankin score, and r = 0.44, p = 0.02 with safranin-O). No significant correlation was found between the histologic score and MMP-13 or cathepsin-K cell score.

## Discussion

This study brings to light some new and interesting information about the mechanisms by which licofelone could reduce the progression of the structural changes caused by OA. A previous study demonstrated that licofelone, a dual inhibitor of both COXs and 5-LOX, had a protective effect on the structural changes in experimental dog OA [6]; the results of this study indicated that the drug works through the inhibition of the synthesis of IL-1β and MMP-1. The present study aimed to expand our knowledge about the effect of licofelone on other major factors involved in the *in situ *degradation of OA cartilage macromolecules, including aggrecans and type II collagen. Moreover, we sought to gain new insight into the effect of the drug on the regulation of 5-LOX, one of the main enzymes involved in the synthesis of LTB_4 _by chondrocytes [4,24].

The results of the study demonstrate that licofelone was very effective at reducing the synthesis of cathepsin K and MMP-13, two highly potent enzymes involved in the *in situ *degradation of type II collagen in OA cartilage. The role of cathepsin K in OA pathophysiology has been previously well documented [14,18,25]. This enzyme has been found not only to be involved in hyalin cartilage degradation, but also likely to be responsible for the resorption of the calcified cartilage and subchondral bone in the early phase of the disease [18]. Licofelone treatment was shown to reduce the level of synthesis of cathepsin K in both of these tissues in experimental dog OA, which may at least partially explain the effects of the drug. The exact mechanism(s) by which licofelone reduces the mRNA expression level of cathepsin K is not fully understood; it is under exploration. Because the synthesis of cathepsin K has been demonstrated to be upregulated by proinflammatory cytokines such as IL-1β and tumor necrosis factor-α [26], however, the capacity of licofelone to inhibit the synthesis of IL-1β [6-8] may explain, at least in part, the effect of the drug on the synthesis of cathepsin K.

Licofelone treatment was also shown *in vitro *to reduce the mRNA expression and protein synthesis of MMP-13 in OA chondrocytes [18,23]. A previous study demonstrated that it also reduces *in situ *the level of this enzyme in OA calcified cartilage and subchondral bone [18], which might explain its effect of inhibiting the remodeling of these tissues. The exact mechanisms by which licofelone could reduce MMP-13 expression in OA chondrocytes have been extensively explored, giving rise to a number of interesting hypotheses. The inhibitory effect of licofelone on the synthesis of IL-1β by the OA synovium is likely to be an important contributing factor [6-8]. In addition, a recent study from our laboratory has demonstrated that the drug's most likely mode of action on MMP synthesis is the inhibition of major intercellular signaling pathways. Experiments have shown that licofelone can inhibit the mRNA expression of IL-1β induced MMP-13 in OA chondrocytes, and that this effect is mediated by the selective inhibition of the activation of the p38 pathway and the downstream transcription factor cyclic-AMP responsive element binding protein (CREB) [23]. This effect is of prime importance for better understanding the mechanisms by which this drug can exert its positive effect on the progression of OA structural changes. MMP-13, or collagenase-3, can cleave both type I and type II collagen; however, the enzyme has a higher degrading activity on type II collagen and can also degrade the aggrecan core protein. Therefore, the inhibition of MMP-13 synthesis in OA chondrocytes by licofelone could explain the drug's positive effect of protecting the cartilage matrix macromolecules that contain predominantly type II collagen and aggrecan. Similarly, the inhibition of MMP-13 synthesis by bone cells and osteoclasts could also exert a positive effect by reducing the extent of the degradation of type I collagen in the subchondral bone matrix [18].

Aggrecans are large aggregating proteoglycans that fill the interstices of the collagen meshwork and give the cartilage its ability to resist compressive loads. MMPs are considered among the main enzymes involved in the degradation of aggrecans in cartilage [15-17,27,28]. Many MMPs that can degrade aggrecans have been demonstrated to be overexpressed in OA cartilage. Of most interest is the recent discovery of a group of enzymes of the ADAMTS family named aggrecanases, which cleave the aggrecan core protein and are believed to play a determinant role in arthritis in the breakdown of aggrecan [15]. Three aggrecanases have thus far been identified: ADAMTS-1, ADAMTS-4, and ADAMTS-5. ADAMTS-1 and ADAMTS-5 are two enzymes that are constitutively expressed in both normal and OA cartilage. There are conflicting reports about the factors regulating the synthesis of these enzymes [15,29,30]. Moreover, the synthesis of these two enzymes in chondrocytes does appear to be variably regulated by IL-1β; in fact, this cytokine might, under certain conditions, downregulate ADAMTS-1 synthesis [31]. The results of a number of studies indicate that the regulatory mechanisms of ADAMTS-4 and ADAMTS-5 gene expression and protein synthesis are complex and may vary based on species and culture conditions [15]. The present study found the level of expression of ADAMTS-5 to be detectable in both normal and OA cartilage, with a somewhat higher, yet variable, level in OA cartilage; however, its level of synthesis in OA cartilage was demonstrated to be significantly increased. Moreover, a diffusion of the enzyme in the OA matrix as shown by immunostaining is in support of this enzyme being involved *in situ *in cartilage matrix macromolecule degradation. Additional support for this hypothesis is also provided by the positive correlation between the immunohistological score of ADAMTS-5 and safranin-O staining in OA cartilage. Dogs treated with licofelone showed a decrease in this level that did not, however, reach statistical significance. Nevertheless, based on recent studies demonstrating the predominant role of ADAMTS-5 in OA cartilage degradation [32,33], it is likely that the latter finding has real significance. Our data are in line with previous studies that demonstrated great variability in the response of chondrocytes to the synthesis of ADAMTS-5 upon stimulation by cytokines and growth factors under actual conditions [15]. A regulation of the enzyme synthesis and activity at the post-transcriptional and/or post-translational level is possible; moreover, it is obvious that the synthesis of ADAMTS-5 *in situ *is likely the result of the combination of stimulation by multiple factors. These, in addition to IL-1β, may include such factors as oncostatin M and transforming growth factor-β, which have been demonstrated to strongly upregulate the genetic expression of aggrecanase in chondrocytes [34] and synovial fibroblasts [35].

Our data on ADAMTS-4 are in line with previous reports demonstrating that the level of this enzyme is very low in normal cartilage but that mRNA expression/protein synthesis of the enzyme is upregulated in OA cartilage [16,17,28,29,36], which supports its likely implication in the pathophysiology of the disease [15]. Similar to ADAMTS-5, our data on ADAMTS-4 demonstrate the presence of matrix staining and a positive correlation between the enzyme level and safranin-O staining, also strongly supporting the hypothesis of its role in OA. Licofelone treatment significantly inhibited the synthesis of the enzyme at the transcriptional level in a dose-dependent manner. The inhibition of both aggrecanases by licofelone also provides strong support for the hypothesis that the drug exerts its protective effect through the inhibition of major pathways involved in OA structural changes. The inhibition of aggrecanases could very well contribute to the net decrease in the loss of cartilage matrix and exert a positive effect on cartilage degradation. This result was confirmed and supported by the positive and significant correlation found with both safranin-O staining and the Mankin score.

The level of 5-LOX was found to be significantly increased in OA compared to normal cartilage. Previous studies have demonstrated that LTB_4 _is a potent factor responsible for upregulating the synthesis of IL-1β [7,8,10,24]. Moreover, both in OA chondrocytes and synovial membranes, the upregulation of IL-1β synthesis by LTB_4 _was responsible for inducing the synthesis of MMPs [8,10,24]. Therefore, it becomes obvious that the increased level of 5-LOX with the subsequent upregulation in IL-1β production in OA tissues could very well play a determining role in the degradation of OA cartilage, first by its local action on chondrocytes and the synthesis of catabolic factors and, second, by being an important upregulating factor in the synthesis of IL-1β by OA synovium. This concept is also supported by the positive correlation found between the 5-LOX cell score and both safranin-O staining and the Mankin score. Therefore, the downregulating effects of licofelone on the level of mRNA expression/protein synthesis of 5-LOX could provide an explanation of how the drug can reduce the synthesis of MMP-13 and ADAMTS-4 and ADAMTS-5 in cartilage [24] as well as the synthesis of IL-1β in synovium. These results also support the possible role of LTB_4 _itself in the autocrine regulation of 5-LOX gene expression.

## Conclusion

This study provides evidence that licofelone treatment in the OA experimental dog model markedly reduces the mRNA expression/protein synthesis of key enzymes involved in the destruction of major cartilage matrix macromolecules, such as type II collagen and aggrecans. These findings provide additional information about the possible mechanisms of action of this drug on OA structural changes.

## Abbreviations

ABC = avidin-biotin complex; ACL = anterior cruciate ligament; ADAMTS = a disintegrin and metalloproteinase with thrombospondin motifs; COX = cyclooxygenase; C_t _= threshold cycle; GAPDH = glyceraldehyde-3-phosphate dehydrogenase; IL = interleukin; LOX = lipoxygenase; LTB_4 _= leukotriene-B_4_; MMP = matrix metalloproteinase; NSAID = non-steroidal anti-inflammatory drug; OA = osteoarthritis; PBS = phosphate buffered saline.

## Competing interests

JPP received support from Merckle GmbH, who manufactures Licofelone, and SL is a consultant for Merckle GmbH. JPP, SL, and JMP are co-authors of DMOAD patent applicate of Licofelone with Merckle GmbH.

## Authors' contributions

JPP, CB, JB, PR, SL, DL and JMP contributed to the study design. JPP, CB, MB, JB, FM, CG and JMP performed the acquisition of data. JPP, CB, FM, PR, DL and JMP analyzed and interpreted the data. JPP, CB, PR, DL and JMP prepared the manuscript. JPP, CB, JB and JMP performed the statistical analysis. All authors read and approved the final manuscript. JPP and CB contributed equally to this manuscript.

## Figures and Tables

**Table 1:** Primer design for quantitative RT-PCR analysis

mRNA	Primers^a^
MMP-13	Fw: 5'-TTGGTCAGATGTGACACCTC Rv: 5'-ATCGGGAAGCATAAAGTGGC
Cathepsin K	Fw: 5'-AGGTGGATGAAATCTCTCGG Rv: 5'-TTCTTGAGTTGGCCCTCCAG
5-LOX	Fw: 5'-TGCGTTCCAGTGACTTCCAC Rv: 5'-CTCTGCACCATCTGCACGTG
ADAMTS-4	Fw: 5'-TACTACTATGTGCTGGAGCC Rv: 5'-AGTGACCACATTGTTGTATCC
ADAMTS-5	Fw: 5'-GGCATCATTCATGTGACAC Rv: 5'-GCATCGTAGGTCTGTCCTG
GAPDH	Fw: 5'-AGGCTGTGGGCAAGGTCATC Rv: 5'-AAGGTGGAAGAGTGGGTGTC

^a^Fw, forward; Rv, reverse. GAPDH, glyseraldehyde-3-phosphate dehydrogenase; MMP, matrix metalloproteinase.
